# Association of Clinical Pathologists, Wessex Branch

**Published:** 1984-01

**Authors:** 


### Association of Clinical Pathologists Wessex Branch

Autumn Meeting, Frenchay Hospital, Bristol, 19th November 1983

### THE ARTERIAL SUPPLY OF HUMAN LYMPH NODES

**D. Semeraro and J. D. Davies, Royal Infirmary, Bristol**

Since Calvert's studies in the late 19th century, the typical human lymph node is depicted with a central hilum through which pass its arteries, veins and efferent lymphatic channels. Subsequently there has been no formal confirmation as to whether this vascular arrangement is in fact a feature of all human lymph nodes. Our study was designed to reinvestigate this traditional view of lymph nodal vascular supply.

The arterial tree supplying lymph nodes from human cadavers was injected with a radio-opaque barium suspension (75% w/v). Superficial lymph nodes from the groin and deep lymph nodes from the small intestinal mesentery were studied by means of specimen radiography and histological examination. Such combined techniques allowed demonstration of the vessels injected, and study of their macoscopic and microscopic distribution.

The superficial lymph nodes from 17 subjects all showed that individual nodes were supplied by a single arterial branch which entered the hilum in the manner described by Calvert. By contrast the deep mesenteric lymph nodes, which usually lack a central hilum, were each supplied in all 8 subjects by two to five arteries which impinge upon them radially from several directions, and from different vascular arcades.

Such marked differences in the arterial supply of superficial and deep lymph nodes probably reflect microanatomical differences in the two types of lymph node. The radial arterial supply of deep nodes appears related to their vascular trabeculae, the more complex arrangement of cortex and paracortex, and to functional characteristics which are features of these lymph nodes. Lack of knowledge of the arterial supply of deep lymph nodes in man has led to misconceptions in the understanding of both reactive and pathological conditions affecting these structures.

### MESENTERIC LYMPH NODE ISCHAEMIA IN VOLVULUS

**N. J. Mahy and J. D. Davies, Royal Infirmary, Bristol**

Perhaps on account of the rich and complex vascular supply of lymph nodes, ischaemia has not received much attention in these organs. Spontaneous lymph nodal infarction may affect normally reactive nodes, although American studies have suggested that nodal infarction usually betokens involvement by malignant lymphoma.

We reported 10 cases of colonic or small intestinal volvulus in which mesenteric lymph nodes were available for histological examination between 1975 and 1983. Lymph nodes displayed frank infarction in 4 of 6 cases in which the bowel showed mucosal necrosis as a result of the volvulus. None of 4 cases of volvulus with entirely viable mucosa showed nodal necrosis. Common to both these groups were lesser lymph nodal ischaemic changes in the form of lymphoidal cell depletion and a distinctive form of capsular hypervascularity. Such lesions were not seen in control patients with a variety of bowel pathology although sinus distention, erythrocyte extravasation and generalised vasodilatation were seen in lymph nodes from cases of volvulus and in other intestinal diseases. No case showed a malignant lymphoma.

Surprisingly this is the first documentation of lymph nodal ischaemia associated with an identifiable vascular lesion causing ischaemic damage in an organ supplied by the same blood vessels. We believe lymph nodal ischaemia to be much more common than is generally realised, and do not feel that the association with malignant lymphoma is as marked as has been suggested.

### EXTRAPULMONARY OAT CELL CARCINOMA

**Nassif B. N. Ibrahim, James C. Briggs and Catherine M. Corbishley**
**Frenchay Hospital, Bristol**

Primary extra pulmonary tumours with histologic features indistinguishable from bronchogenic oat cell carcinoma are appearing with increasing frequency in the literature. These tumours have been described in the esophagus, stomach, pancreas, larynx, hypopharynx, salivary glands, nasal cavity and paranasal sinuses, thymus, small and large bowel, uterine cervix, endometrium, breast, prostate, urinary bladder and skin. It is now widely believed that oat cell carcinoma is a poorly differentiated counterpart of carcinoid tumour and that both originate from an endocrine cell system. We reviewed all cases of extra pulmonary oat cell carcinomas, which we were able to find in the English literature, and reported personally studied examples of these tumours, occurring in the esophagus, stomach and urinary bladder. A closely related, if not identical, tumour arising in the skin was also described. We would emphasise that a wider recognition of these tumours is likely to lead to their more frequent diagnosis and possible treatment.

### THE CLASSIFICATION OF BRAIN LYMPHOMA USING MONOCLONAL ANTIBODIES

**P. M. Allan, H. B. Coakham, E. I. Harper and B. Brownell, Frenchay Hospital, Bristol**

Monoclonal antibodies have been particularly useful in both the biological characterisation and differential diagnosis of systemic lymphomas, but primary brain lymphomas have not as yet been classified in the same detail as their systemic counterparts. We have therefore examined 10 brain lymphomas with a panel of 10 monoclonal antibodies, including markers for B cells, B cell progenitors, T cells, T cell subsets, myeloid series cells, in addition to routine histology.

In 4 of the cases, routine histological techniques were not diagnostic and immunocytochemistry was responsible for making the diagnosis. In the 10 cases of intracranial lymphoid tumour, 8 were shown to be B cell lymphomas with no detectable systemic involvement, one was a myeloid deposit and one a hairy cell leukaemia deposit.

Our findings suggest that primary brain lymphomas are B cell lesions, and their place in the current biological classification of lymphoid malignancies will be discussed, as will the clinical implications of accurate diagnosis of this disease.

### THE ISOLATION OF AEROMONAS SP. FROM THE FAECES OF PATIENTS WITH DIARRHOEA

**Joy Harrison, A. Bornemisza and P. Thomas, Frenchay Hospital, Bristol**

The routine methods used by most laboratories for screening faeces for enteric pathogens generally fails to identify Aeromonas species. It is usually dismissed as not significant along with other lactose/sucrose fermenting organisms. If the strain does not ferment lactose/sucrose it is not identified by the range of biochemical screening tests generally used. On the grounds of simplicity, cost and success, Frenchay Hospital Laboratory is now using a modification of the method of Shread et al (1981). Faeces are initially inoculated into alkaline peptone water, incubated at 30°C overnight and subcultured onto xylose deoxycholate agar, and incubated overnight at 37°C. Non-xylose-fermenting colonies which give a positive oxidase test are identified by API.10 s. The majority of strains are biotyped by a short range of additional biochemical tests.

### RESULTS

Examination of faeces from 1097 patients with diarrhoea during a one-year survey yielded the results shown in the following Table

**Table**

**Enteric pathogens isolated from 1097 patients with diarrhoea during a 1-year study**

| *Organism* | *Total* | *Abroad* | *G.B.* |
|---|---|---|---|
| Aeromonas | 43 | 4 | 39 |
| Campylobacter | 50 | 13 | 37 |
| Salmonella | 23 | 8 | 15 |
| Shigella | 3 | 2 | 1 |
| Path. *E. Coli* | 9 | 0 | |
| Parasites | 13 | 5 | |
| Vibrio | 0 | 0 | |

Sera from 13 patients were tested to see whether agglutination was given with strains of Aeromonas isolated from their own faeces. Two sera gave titres of 1 in 80 and the remainder 1 in 20 or less. Fifty normal control sera from N.B.T.S. were also tested against 18 strains of Aeromonas and 2 of these gave titres of 1 in 40 and the rest 1 in 20 or less. It was concluded that serum agglutination tests did not yield useful diagnostic results.

### CONCLUSION AND FUTURE WORK

Our year long study has shown that Aeromonas is a highly significant cause of diarrhoea in U.K. residents as well as in overseas travellers, second only to Campylobacter in frequency. The condition is often a short self-limiting disease but may occasionally cause chronic diarrhoea lasting several months. A serological test would be helpful in assessing the significance of the isolation of Aeromonas from the stools of a patient with chronic diarrhoea. Wadstrom et al. (1976) showed the presence of three toxins: haemolysin, cytotoxin and an enterotoxin; the latter having the same activity to that of V. cholerae. Burke (1982) found a 97% correlation between haemolysin and enterotoxin production. Work is therefore being carried out in our laboratory on an antihaemolysin test to detect antibodies to Aeromonas in patients with Aeromonas enteritis.

### MECHANISMS IN THE INDUCTION OF FEVER

**J. T. Whicher, Royal Infirmary, Bristol**

Fever is an adaptive response to inflammation shown by all homoiotherms and also by poikilotherms such as reptiles. A considerable body of evidence suggests that it enhances a number of the cellular responses of inflammation and increases survival from infection. It is mediated by the monokine interleukin I and forms part of a group of mechanisms potentiating inflammation which include the acute phase response. These responses may be defective in some chronic inflammatory diseases and possibly play a part in pathogenesis.

### CLINICAL CHEMISTRY AND HAEMATOLOGY NEARER THE PATIENT

**R. D. Eastham, Frenchay Hospital, Bristol**

At an International Conference at Surrey University (September 1983), the possible effects of recently developed laboratory apparatus were discussed. Relatively small, cheap, easily used pieces of equipment are now on the market for use in the side ward, Intensive Care Unit, Operating Theatre, and possibly Health Centre. These would enable the clinician to obtain the results of certain tests rapidly, without the necessity of sending blood or urine samples to the Department of Pathology. In particular, blood glucose, plasma $PO_2$, $PCO_2$, pH, bicarbonate, plasma sodium and potassium, plasma and urine osmolality, haemoglobin, white count and platelet counts, could all be estimated outside the laboratory with apparent accuracy and precision.

Problems discussed included: (1) Which budget pays for the initial purchase, increased running costs, maintenance and repairs, if the apparatus is not directly under the control of the Laboratory? (2) Who is responsible for day-to-day maintenance and quality control? (3) Methods of assessment of the 'black box' apparatus in comparison with 'main frame' apparatus in the Laboratory. (4) Who uses the new equipment? Nurses, and/or clinical doctors, and/or technicians, and/or MLSO's seconded from the Laboratory? (5) What are the implications of Safety at Work and the Howie Report?

The problems arising are directly in proportion to the distance between the main Laboratory and the site of the new equipment, and to the speed with which clinicians require certain test results.

### THE CAUSE OF SOME OF THE EFFECTS OF POTASSIUM DEPLETION ON THE RENAL TUBULAR CELL IS INCREASED AMMONIA PRODUCTION

**Denis St J. O'Reilly, Royal Infirmary, Bristol**

Potassium depletion is known to cause vacuolation of renal tubular cells which is associated with defects in renal tubular function, polyuria and resistance to vasopressin, tubular proteinuria and an increase in the urinary excretion of the lysosomal enzyme N-Acetyl-β-Glucosaminidase (NAG). Potassium depletion also causes an increase in ammonia production by renal tubular cells. The effects of an increase in intracellular ammonia can explain the above changes due to potassium depletion on renal tubular cell function and morphology.

### THE VALUE OF RENAL IMMUNOFLUORESCENCE IN NECROPSY MATERIAL

**G. S. Reynolds, J. M. Poulding and C. R. Tribe, Southmead Hospital, Bristol**

Renal immunofluorescence (I.F.) is of proven value to the clinician as a routine procedure in diagnostic renal biopsy. It might also be of value to the pathologist at necropsy in 5 situations:

1. Sudden death with a possible renal cause, e.g. hypertensive cerebral haemorrhage;
2. Some forensic cases, e.g. maternal death with undiagnosed pre-eclampsia;
3. Chronic renal failure without biopsy-proven disease;
4. Follow-up of biopsy-proven renal disease to study natural history and/or the effects of treatment;
5. As a research tool screening for occult renal disease in necropsy series.

To be valid, post-mortem renal I.F. findings must be similarly interpretable to those in life. It must be shown that there is similar uptake of antibodies, that effects due to death itself and to the interval between death and necropsy are negligible or predictable, and that findings after death are congruent with those in a patient's previous biopsies.

Post-mortem renal I.F. was done in 84 cases in Bristol between 1974 and mid-1983, drawn from Southmead Hospital (67), Bristol City Mortuary (16), and Bristol Children's Hospital (1). 15 patients had also had at least one renal biopsy with I.F. A standard panel of 5 direct fluorescent antibodies was used, against IgG, IgA, IgM, fibrin/fibrinogen and complement (C3). The interval between biopsies and death ranged from 2 days to just under 4 years, and that between death and necropsy from 2 hours to 5 days. Standard mortuary refrigerators were used for storage.

We found that with immunoglobulins, post-mortem renal I.F. findings in a given disease were the same as in life or showed expected progression. The same was true for complement. Death did not by itself cause the deposition of immunoglobulins in kidney. This was less so with complement, there being some apparent 'false positive' results. The findings for fibrin/fibrinogen were unpredictable and inconsistent.

We therefore conclude that post-mortem renal I.F. is an interpretable, reliable procedure yielding useful and consistent results.

